# Review of the genus *Vekunta* Distant from China, with descriptions of two new species (Hemiptera, Fulgoromorpha, Derbidae)

**DOI:** 10.3897/zookeys.825.31542

**Published:** 2019-02-19

**Authors:** Yong-Jin Sui, Xiang-Sheng Chen

**Affiliations:** 1 Institute of Entomology, Guizhou University, Guiyang, Guizhou, 550025, China Guizhou University Guiyang China; 2 Special Key Laboratory for Development and Utilization of Insect Resources of Guizhou, Guizhou University, Guiyang, Guizhou, 550025, China Guizhou University Guiyang China; 3 Guizhou Key Laboratory for Plant Pest Management of Mountainous Region, Guizhou University, Guiyang, Guizhou, 550025, China Guizhou University Guiyang China

**Keywords:** Cenchreini, distribution, Fulgoroidea, planthoppers, taxonomy

## Abstract

The derbid genus *Vekunta* Distant, 1906 is reviewed. Two new species, *V.bambusana***sp. n.** and *V.pentaprocessusa***sp. n.**, are described and illustrated from the southwest of China to give the genus twenty-nine species in China. A checklist and a key to species of the genus from China are also provided.

## Introduction

The planthopper family Derbidae (Hemiptera, Fulgoromorpha) was established by Spinola in 1839, containing three subfamilies in twenty tribes (Bourgoin 2018). Approximately eight tribes, 38 genera, and 154 species of Derbidae are known in China. Almost all members of the family in China are distributed in the Oriental region, especially in southern China.

The planthopper genus *Vekunta* was established by Distant (1906b) with two species from Sri Lanka and with *Temesatenella* Melichar, 1903 as its type species. This genus belongs to the tribe Cenchreini of subfamily Derbinae (Hemiptera: Derbidae). The tribe Cenchreini was established by Muir in 1913, containing 23 genera and 185 species to date, is a larger tribe of Derbidae (Bourgoin 2018). Characteristics of the tribe Cenchreini are the forewing with clavus closed rarely open; clavus at least half as long as whole forewing; MP and CuA usually with less than eight branches at margin; Pcu on clavus (forewing) with sensory pits; hindwing more than half as long as forewing; frons narrow, usually not strongly compressed (Fennah 1952, Emeljanov 1996). The genus *Vekunta* is the largest one of Cenchreini (Hemiptera: Derbidae); 43 species have hitherto been recorded in this genus (Bourgoin 2018), including 27 species from China (Matsumura 1914, 1940; Muir 1914; Fennah 1956; Yang and Wu 1993; Liang and Wu 2001), seven from Indonesia (Walker 1857; Bierman 1910; Muir 1913, 1915, 1923, 1926; Liang 2000), two from Philippines (Melichar 1914; Muir 1917), two from India (Muir 1922; Liang and Wu 2001), two from Korea (Rahman et al. 2012), one from Japan (Matsumura 1914), one from Vietnam (Fennah 1978), one from Papua New Guinea (Walker 1870; Liang 2000), and one from the Seychelles (Löcker et al. 2009).

Herein, two new species, *Vekuntabambusana* sp. n. and *V.pentaprocessusa* sp. n., are described and illustrated from Guizhou and Yunnan provinces, China. A checklist and a key to species of the genus from China are also provided.

## Materials and methods

The morphological terminology follows Bourgoin (1987) and Yang and Wu (1993). The morphological terminology of female genitalia follows Bourgoin (1993). Body length was measured from apex of vertex to tip of forewing. The standard terminology of venation follows Bourgoin et al. (2015). The term “anal style” used here follows Rahman et al. (2012). Dried specimens were used for the description and illustration. External morphology was observed under a stereoscopic microscope and characters were measured with an ocular micrometer. Color pictures for adult habitus were obtained by the Nikon SMZ25 system. The genital segments of the examined specimens were macerated in 10% NaOH and drawn from preparations in glycerin jelly using a Leica MZ 12.5 stereomicroscope. Illustrations were scanned with a Canon CanoScan LiDE 220 and imported into Adobe Photoshop CS5 for labeling and plate composition. The dissected genitalia were preserved in glycerin in small plastic tubes pinned together with the specimens.

The type specimens are deposited in the Institute of Entomology, Guizhou University, Guiyang, Guizhou Province, China (**GUGC**).

## Taxonomy

### Genus Vekunta

Taxon classificationAnimaliaHemipteraDerbidae

Distant, 1906

Figs 1–4 , 5–14 , 15–19 , 20–29 , 30–34

Temesa Melichar, 1903: 40; preoccupied by Temesa (Mollusca) Adams, 1855.
Vekunta Distant, 1906a: 8; 1906b: 287; Yang and Wu 1993: 97; Liang and Wu 2001: 511–512; Löcker et al. 2009: 15; Rahman et al. 2012: 24.

#### Type species.

*Temesatenella* Melichar, 1903 by original designation.

#### Diagnosis.

**Figures 1–4.**
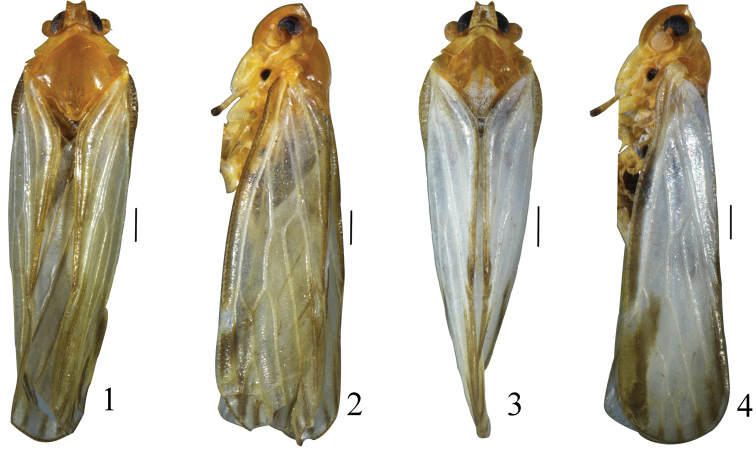
Male habitus (dorsal and lateral view). **1–2***Vekuntabambusana* sp. n. **3–4***Vekuntapentaprocessusa* sp. n. Scale bar: 0.5 mm.

**Figures 5–14.**
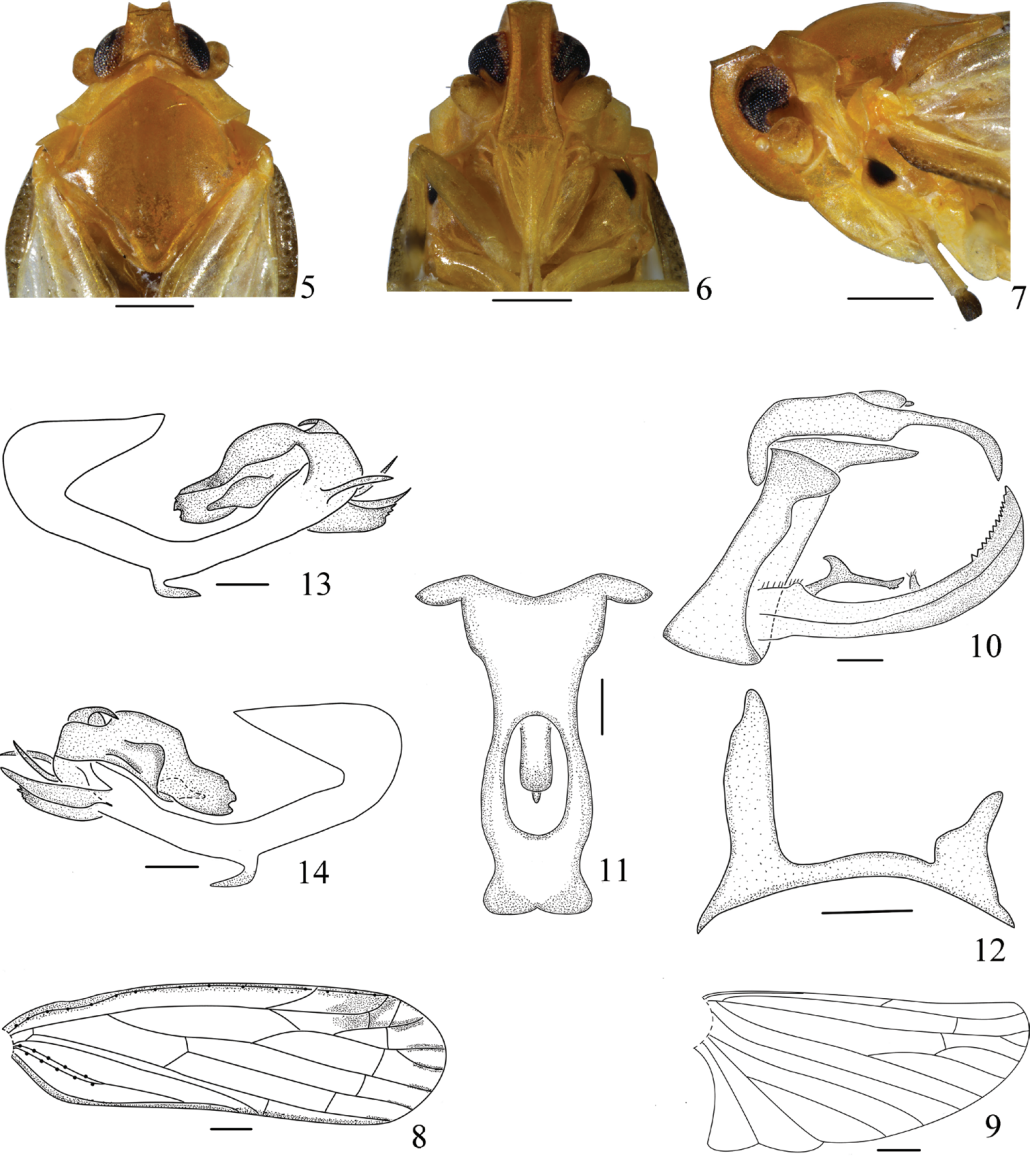
*Vekuntabambusana* sp. n., male. **5** Head and thorax, dorsal view **6** face **7** head and thorax, left lateral view **8** forewing **9** hindwing **10** genitalia, left lateral view **11** anal tube, dorsal view **12** dorsocaudal processes of pygofer, dorsal view **13** phallus, left lateral view **14** phallus, right lateral view. Scale bars: 0.5 mm (**5–7**); 0.2 mm (**8–14**).

Combination of the following characters: head (Figs 2, 4, 7, 22) in profile distinctly angulate. Vertex (Figs 1, 3, 5, 20) quadrate, at base wider than at apex, slightly projecting in front of eyes, covered with sensory pits, divided from frons by transverse carina. Frons (Figs 6, 21) without median carina, elongate to quadrate. Postclypeus with three carinae. Antennae (Figs 5–7, 20–22) short, second antennomere oval, subantennal process (Figs 6, 7, 21, 22) small or absent. Ocelli (Figs 7, 22) present. Forewing (Figs 8, 23) with short subcostal cell, Sc+R fused with MP for a short distance, forking nearly basal one-fifth, MP with two sectors, CuA two branched, forking nearly basal one-third, costal margin and vein Pcu covered with tubercles, Pcu+A1 reaching forewing margin near middle. Hindwing (Figs 9, 24) shorter than forewing, MP two branched, forking apically, CuA three branched, forking near middle, CuP and Pcu single, A1 two branched. Spinal formula of hind leg 7–6–6.

### Checklist of species of *Vekunta* Distant, 1906 from China

*V.albipennis* Matsumura, 1914; China (Taiwan)

*V.asymmetrica* Liang & Wu, 2001; China (Xizang)

*V.atripennis* Matsumura, 1940; China (Taiwan)

*V.bambusana* sp. n.; China (Guizhou)

*V.botelensis* Matsumura, 1940; China (Taiwan)

*V.commendata* Yang & Wu, 1993; China (Taiwan)

*V.diluta* Yang & Wu, 1993; China (Taiwan)

*V.extima* Yang & Wu, 1993; China (Taiwan)

*V.fera* Yang & Wu, 1993; China (Taiwan)

*V.gracilenta* Yang & Wu, 1993; China (Taiwan)

*V.intermedia* Yang & Wu, 1993; China (Taiwan)

*V.kotoshonis* Matsumura, 1940; China (Taiwan)

*V.lyricen* Fennah, 1956; China (Taiwan)

*V.maculata* Matsumura, 1914; China (Taiwan)

*V.makii* Muir, 1914; China (Taiwan)

*V.malloti* Matsumura, 1914; China (Taiwan), Japan (Honshu, Kyushu, Shikoku)

*V.memoranda* Yang & Wu, 1993; China (Taiwan)

*V.nigra* Yang & Wu, 1993; China (Taiwan)

*V.nigrolineata* Muir, 1914; China (Taiwan)

*V.nivea* Fennah, 1956; China (Zhejiang)

*V.nutabunda* Yang & Wu, 1993; China (Taiwan)

*V.obaerata* Yang & Wu, 1993; China (Taiwan)

*V.obliqua* Yang & Wu, 1993; China (Taiwan)

*V.parca* Yang & Wu, 1993; China (Taiwan)

*V.pentaprocessusa* sp. n.; China (Yunnan)

*V.shirakii* Matsumura, 1914; China (Taiwan)

*V.stigmata* Matsumura, 1914; China (Taiwan)

*V.triprotrusa* Wu & Liang, 2001; China (Yunnan)

*V.umbripennis* Muir, 1914; China (Taiwan)

### Key to species of the genus *Vekunta* Distant from China (based on Rahman et al. 2012)

**Table:** 

1	Thorax with propleura with a large dark spot	*** V. albipennis ***
–	Thorax with propleura not as above	**2**
2	Forewing along costal and anal margins with brown to dark brown stripe	**3**
–	Forewing along costal and anal margins without brown to dark brown stripe	**6**
3	Female sternite VII with protrusion asymmetrical (Yang and Wu 1993: fig. 62E)	*** V. diluta ***
–	Female sternite VII with protrusion symmetrical (Figs 17, 32)	**4**
4	Female sternite VII with protrusion length longer than width at base (Yang and Wu 1993: fig. 64D)	*** V. nigrolineata ***
–	Female sternite VII with protrusion length shorter than width at base (Figs 17, 32)	**5**
5	Male with gonostyli bilaterally symmetrical (Fig. 10); left side of aedeagus with a laminal process near middle, apex of aedeagus valviform, reaching to middle of periandrium (Figs 13–14)	***V.bambusana* sp. n.**
–	Male with gonostyli asymmetrical, right gonostylus larger than left one (Fig. 25); aedeagus with five spinous processes at apex, the largest process produced reaching to basal of periandrium (Figs 28–29)	***V.pentaprocessusa* sp. n.**
6	Forewing yellowish white	**7**
–	Forewing pale brown, dark or with dark markings	**16**
7	Pygofer of male with dorsocaudal processes asymmetrical	**8**
–	Pygofer of male with dorsocaudal processes symmetrical	**9**
8	Aedeagus of male not reaching to middle of periandrium (Yang and Wu 1993: fig. 55H)	*** V. nutabunda ***
–	Aedeagus of male reaching to middle of periandrium (Yang and Wu 1993: fig. 60H–I)	*** V. commendata ***
9	Aedeagus of male with process(es) at base	**10**
–	Aedeagus of male without process at base (Yang and Wu 1993: fig. 52G)	*** V. extima ***
10	Periandrium with 4–5 processes in male	**11**
–	Periandrium with 2 processes in male	**13**
11	Periandrium with 5 processes in male (Yang and Wu 1993: fig. 50G–H)	*** V. maculata ***
–	Periandrium with 4 processes in male	**12**
12	Periandrium of male with one pair of slender processes at base (Yang and Wu 1993: fig. 51H); anal tube almost straight apically (Yang and Wu 1993: fig. 51E)	*** V. makii ***
–	Periandrium of male with one spinous process at base (Fennah 1956: fig. 12D); anal tube abruptly turned downward then cephalad apically (Fennah 1956: fig. 12E)	*** V. nivea ***
13	Male with apical part of anal tube strongly curved in lateral profile	**14**
–	Male with apical part of anal tube slightly curved in lateral profile	**15**
14	Periandrium of male with 2 short processes near middle, one directed caudally, another one directed dorsally (Yang and Wu 1993: fig. 56H)	*** V. gracilenta ***
–	Periandrium of male with 2 long processes near middle, all directed caudally (Yang and Wu 1993: fig. 59H)	*** V. obliqua ***
15	Male with apical margin of anal tube broadly rounded; periandrium with short process at left base reaching less than middle (Yang and Wu 1993: fig. 57G)	*** V. intermedia ***
–	Male with apical margin of anal tube truncate obliquely; periandrium with long process at left base reaching over than middle (Yang and Wu 1993: fig. 58G)	*** V. obaerata ***
16	Aedeagus of male, in right lateral view, with a small process near base and another lobe-like process in the middle (Yang and Wu 1993: fig. 53H)	*** V. parca ***
–	Aedeagus of male not as above	**17**
17	Forewing with scattered dark markings	**18**
–	Forewing uniformly dark except stigma	**22**
18	Mesothorax pale yellow	**19**
–	Mesothorax fuscous or brown	**20**
19	Pygofer of male with symmetrical dorsocaudal processes; periandrium with one pair of stout processes at base (Yang and Wu 1993: fig. 45F)	*** V. lyricen ***
–	Pygofer of male with asymmetrical dorsocaudal processes; periandrium without process at base (Yang and Wu 1993: fig. 46G)	*** V. kotoshonis ***
20	Frons between the lateral carinae reddish yellow	*** V. botelensis ***
–	Frons between the lateral carinae brownish	**21**
21	Forewing veins very dark, paler toward apex	*** V. atripennis ***
–	Forewing veins sordid yellow	*** V. shirakii ***
22	Hindwing black	**23**
–	Hindwing not black	**26**
23	Antennae yellow	*** V. triprotrusa ***
–	Antennae brown	**24**
24	Male with dorsocaudal processes of pygofer triangularly produced (Yang and Wu 1993: fig. 63B)	*** V. malloti ***
–	Male with dorsocaudal processes of pygofer not triangularly produced	**25**
25	Aedeagus of male with 2 hooked processes at basoventral portion (Yang and Wu 1993: fig. 47G); male with dorsocaudal processes of pygofer not produced (Yang and Wu 1993: fig. 47E)	*** V. stigmata ***
–	Aedeagus of male without hooked process at basoventral portion (Yang and Wu 1993: fig. 48G); male with dorsocaudal process of pygofer broadly rounded (Yang and Wu 1993: fig. 48E)	*** V. memoranda ***
26	Hindwing white	**27**
–	Hindwing gray	**28**
27	Periandrium of male with a process at base (Yang and Wu 1993: fig. 61I); apex of anal tube curved (Yang and Wu 1993: fig. 61E)	*** V. fera ***
–	Periandrium of male without process at base (Wu and Liang 2001: fig. 19); apex of anal tube straight (Wu and Liang 2001: fig.16)	*** V. asymmetrica ***
28	Pygofer of male with dorsocaudal processes asymmetrical; periandrium without process at base (Yang and Wu 1993: fig. 49G)	*** V. umbripennis ***
–	Pygofer of male with dorsocaudal processes symmetrical; periandrium with one pair of hooked processes at base ventrally and two long processes laterally, one process at base and another in the middle (Yang and Wu 1993: fig. 54H)	*** V. nigra ***

### Vekunta bambusana sp. n.

Taxon classificationAnimaliaHemipteraDerbidae

http://zoobank.org/12B8BE91-60B4-46C7-A7AB-3EF08D50F70A

Figs 1 , 2 , 5–14 , 15–19

#### Type material.

***Holotype*** ♂, CHINA: **Guizhou**, Wangmo, Dayi (25°22'N, 106°06'E), 21 August 2012, Z-M Chang. ***Paratypes***, **Guizhou**: 1♂, Wangmo, Dayi, 23 August 2012, Z-M Chang; 2♂♂, Wangmo, Dayi, 13 August 2014, Z-M Chang; 3♂♂4♀♀, Wangmo, Dayi, 13–14 August 2014, Y Liu; 2♂♂, Suiyang, Wangcao (28°07'N, 107°16'E), 29 July 2014, H-Y Sun; 1♂, Suiyang, Wangcao, 29 July 2014, Y-J Wang.

#### Measurements.

Body length (including forewing): male 5.99–6.37 mm (n = 10), female 6.98–7.03 mm (n = 4); forewing length: male 5.02–5.45 mm (n = 10), female 5.96–6.02 mm (n = 4).

#### Description.

*Coloration.* General color yellow. Head (Figs 1, 2, 5–7) yellow. Vertex (Figs 1, 5) yellow, lateral and apical carinae yellow to brownish yellow. Frons and clypeus yellow (Fig. 6). Rostrum (Fig. 7) yellow with apex fuscous. Gena (Fig. 7) yellow. Eyes (Figs 1, 2, 5–7) black, ocelli yellow. Antennae (Figs 5–7) yellow. Pronotum, mesonotum and tegula yellow (Fig. 5). Forewing (Figs 1, 2) yellowish white except costal and clavus margins from base to near apex brown to dark brown, veins yellowish white. Hindwing subhyaline, yellowish white, veins white. Thorax with ventral areas yellow, mesopleura (Figs 2, 7) with an oval black spot. Legs brownish yellow. Genital segment yellow.

*Head and thorax.* Head (Figs 1, 5) including eyes distinctly narrower than pronotum (1:1.65). Vertex (Figs 1, 5) at base wider than length in middle line (1:0.62), apex narrower than base (1:1.31), straightly projecting before eyes, median carina absent, lateral margin distinctly carinate, posterior margin slightly concave. Frons (Fig. 6) moderately narrow, near frontoclypeal suture widest, disc concave, separated on both sides, subparallel, lateral margin distinctly carinate, median carina absent. Postclypeus (Fig. 6) with median and lateral carinae; anteclypeus with weak median carina, lateral carinae absent. Apical segment of rostrum longer than wide. Antennae (Figs 5–7) short, second antennomere oval, flagellum originated from apical point. Subantennal processes (Figs 6, 7) small. Eyes (Figs 5–7) semicircular; ocelli present, adjacent to eyes. Median length of pronotum short, anterior margin between eyes convex, posterior margin deeply concave, median carina distinct. Mesonotum (Fig. 5) as long as broad, convex, in lateral view raised above vertex, with median and lateral carinae weak, posterior end triangularly depressed. Forewing (Fig. 8) narrow, 3.5 times as long as the widest point, clavus closed, claval veins with a prominent ridge of tubercles, base of costal margin curved inward, costal margin also granulated. Hindwing (Fig. 9) shorter than forewing. Hind tibia without lateral spine.

*Male genitalia*. Anal tube (Fig. 10) in profile broad at basal half, abruptly narrowed medially, apex evenly turned downward, directed ventrally, anal style sets at basal two-fifths; in dorsal view (Fig. 11), length in middle line approximately three times as long as wide at middle, symmetrical, apical margin evenly incised medially. Pygofer (Fig. 10) in lateral view narrowed, dorsocaudal processes (Fig. 12) of pygofer asymmetrical, right dorsocaudal process distinctly longer than left one. Gonostyli (Fig. 10) bilaterally symmetrical, large, elongate and slightly reaching over apex of anal tube in lateral view, dorsal margin serrate at apex, curved dorsally, inner side of laterodorsal margin with a bifurcate process at base and a finger-shaped process medially. Phallus (Figs 13, 14) asymmetrical, periandrium curved, with a hooked process near middle ventrally directed caudally, apex with two spinous processes, below them with two sheet processes, all visible in both left and right lateral view. Aedeagus at base with a process curved dorsally, pointed ventrally, left side of aedeagus with a laminal process near middle, apex of aedeagus valviform, reaching to middle of periandrium.

**Figures 15–19.**
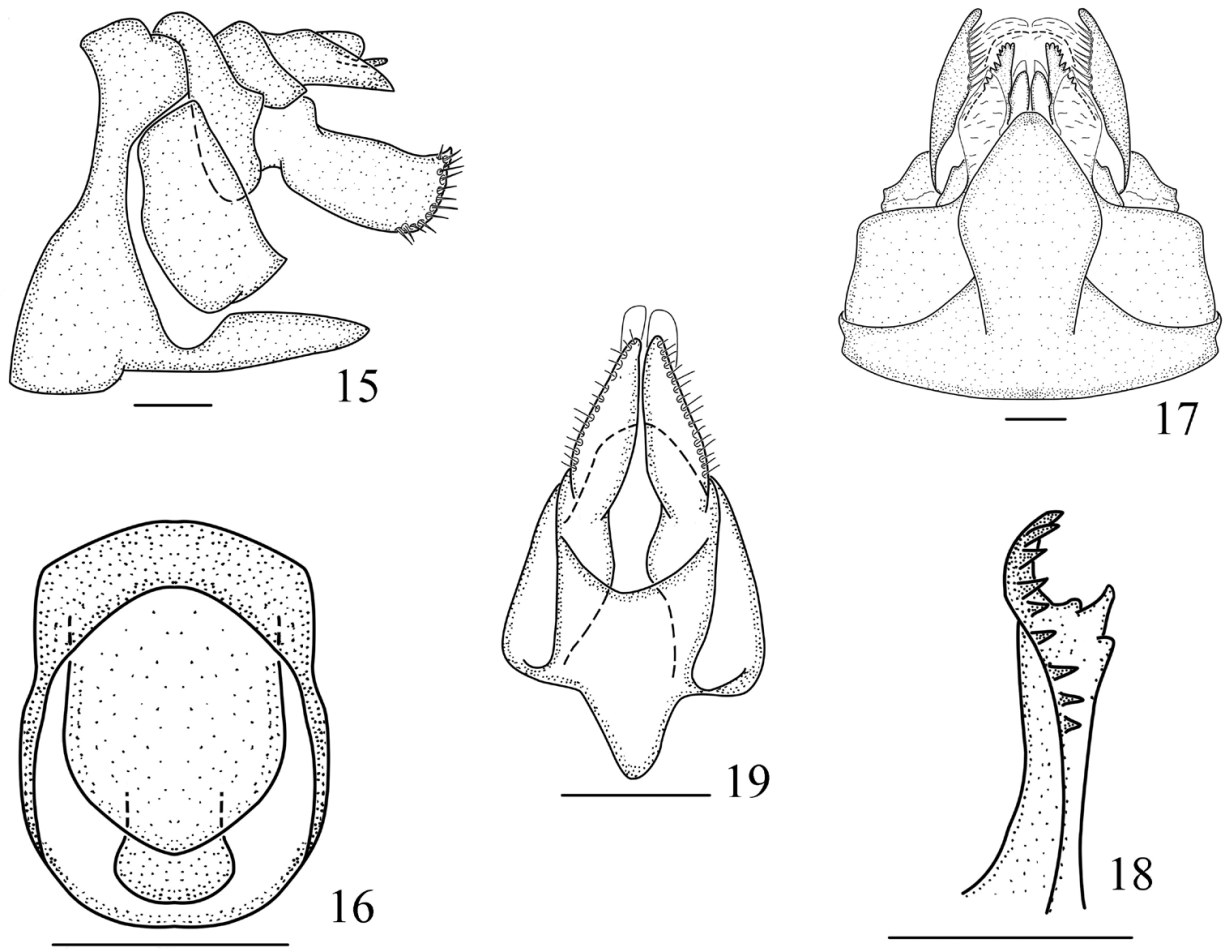
*Vekuntabambusana* sp. n., female. **15** Genitalia, lateral view **16** anal tube, dorsal view **17** genitalia, ventral view **18** gonapophysis VIII, right lateral view **19** gonapophysis IX, ventral view. Scale bar: 0.2 mm.

*Female genitalia.* Anal tube (Figs 15, 16) symmetrical and ring-shaped in dorsal view; apex of anal tube slightly exceeding apex of anal style. Abdominal sternite VII (Fig. 17) in ventral view symmetrical, posterior margin protruded medially, with protrusion length shorter than width at base, lateral margin widened toward the middle and then narrowed gradually toward apex, apical margin rounded. Gonapophysis VIII (Figs 17, 18) with nine teeth at ventral margin. Gonapophysis IX (Fig. 19) with two lobes incompletely symmetrical, lateral margin with dense setae, each lobe with a membrane sheet dorsally, blunt apically. Gonoplac (Figs 15, 17) in lateral view nearly rectangular, with a small angulate process at apex dorsally, lateral margin with spiniform setae.

#### Remarks.

This species is similar to *V.pentaprocessusa* sp. n., but distinguished from the latter by: gonostyli (Fig. 10) symmetrical (gonostyli asymmetrical, with right gonostylus distinctly larger than left one in *V.pentaprocessusa* sp. n.); anal tube (Fig. 11) of male symmetrical in dorsal view (asymmetrical in dorsal view in *V.pentaprocessusa* sp. n.); right dorsocaudal process (Fig. 12) of pygofer in dorsal view distinctly longer than left one in male (left dorsocaudal process in dorsal view slightly longer than right one in *V.pentaprocessusa* sp. n.); periandrium (Figs 13, 14) with a hooked process near middle ventrally (periandrium with a hooked process near base ventrally in *V.pentaprocessusa* sp. n.); aedeagus (Figs 13, 14) valviform at apex, reaching to middle of periandrium (aedeagus with five spinous processes at apex, the largest process reaching to base of periandrium in *V.pentaprocessusa* sp. n.).

#### Etymology.

The species name is derived from the host plant scientific name, Bambusoideae.

#### Host plant.

Bamboo.

#### Distribution.

China (Guizhou).

### Vekunta pentaprocessusa sp. n.

Taxon classificationAnimaliaHemipteraDerbidae

http://zoobank.org/21579E13-C4AB-440D-966E-D8CE49FFB568

Figs 3 , 4 , 20–29 , 30–34

#### Type material.

***Holotype***: ♂, CHINA, **Yunnan**: Mt Gaoligong National Natural Reserve (25°17'N, 98°48'E), light trap, 15 August 2013, Y- J Wang. ***Paratypes***, **Yunnan**: 5♂♂1♀, same date as holotype; 3♂♂, Mt Gaoligong National Natural Reserve, light trap, 13 June 2011, J-K Long; 6♂♂2♀♀, Mt Gaoligong National Natural Reserve, light trap, 13–16 August 2013, W-C Yang, H-Y Sun, Y-J Wang; 1♂, Mt Gaoligong National Natural Reserve, light trap, 12 August 2018, L-J Yang.

#### Measurements.

Body length (including forewing): male 6.17–6.48 mm (n = 16), female 6.96–6.99 mm (n = 3); forewing length: male 5.36–5.40 mm (n = 16), female 6.04–6.11 mm (n = 3).

#### Description.

**Figures 20–29.**
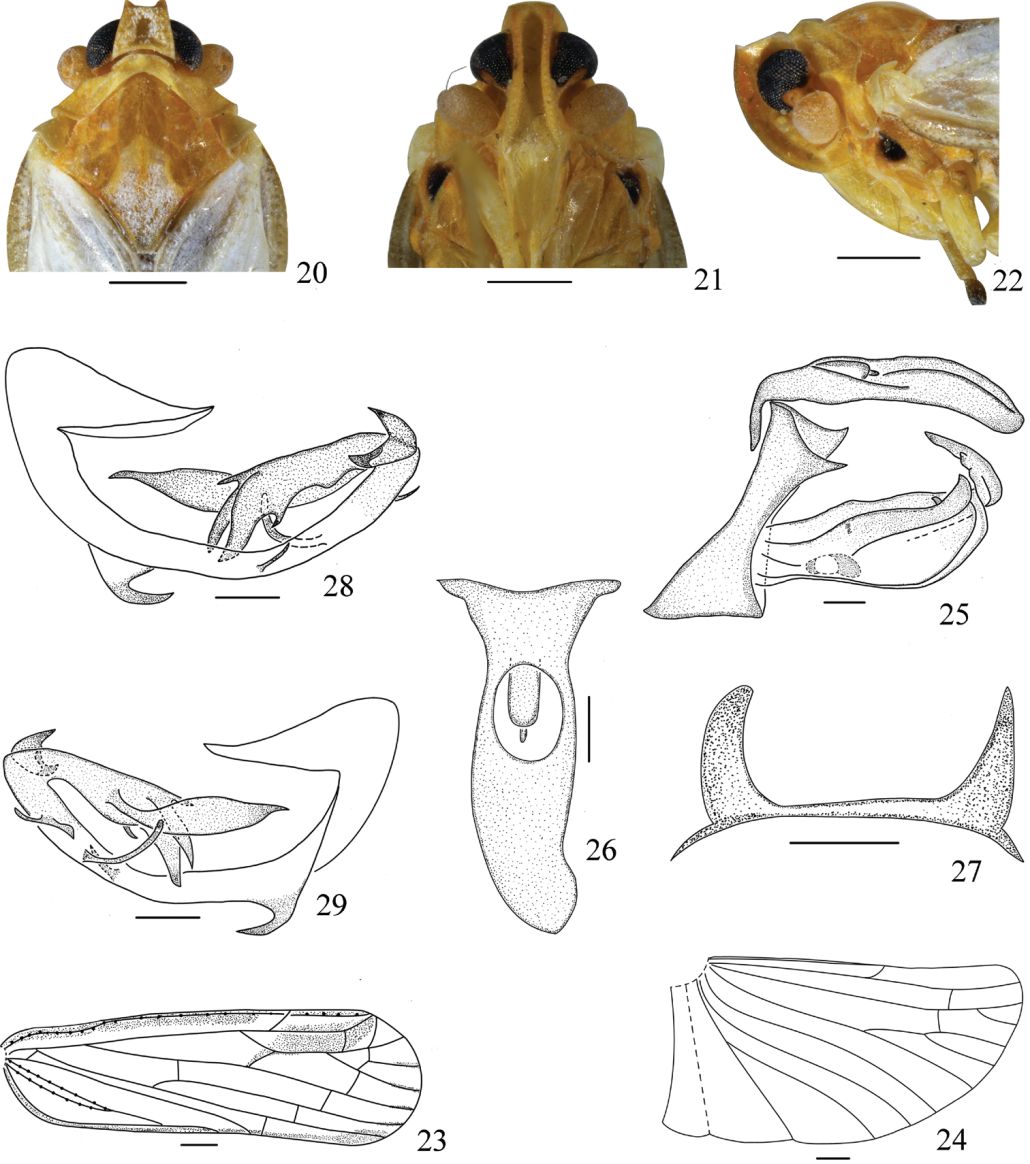
*Vekuntapentaprocessusa* sp. n., male. **20** Head and thorax, dorsal view **21** face **22** head and thorax, left lateral view **23** forewing **24** hindwing **25** male genitalia, left lateral view **26** anal tube of male, dorsal view **27** dorsocaudal processes of pygofer, dorsal view **28** phallus, left lateral view **29** phallus, right lateral view. Scale bars: 0.5 mm (**20–22**); 0.2 mm (**23–29**).

*Coloration.* General color yellow. Head (Figs 3, 4, 20–22) yellow. Vertex (Figs 3, 20) with lateral and apical carinae yellow. Frons (Fig. 21) with lateral margins yellow. Clypeus (Fig. 21), gena (Fig. 22), and antennae (Figs 20–22) yellow. Rostrum yellow with apex fuscous. Eyes (Figs 3, 4, 20–22) black, ocelli yellow. Pronotum, mesonotum and tegula yellow (Figs 3, 20). Forewing (Figs 3, 4) white except with costal and clavus margins from base to near apex brown to dark brown, veins white. Hindwing subhyaline, white, veins white. Thorax with ventral areas yellow, mesopleura (Figs 4, 22) with an oval black spot. Legs pale yellow. Genital segment yellow.

*Head and thorax.* Head (Figs 3, 20) including eyes distinctly narrower than pronotum (1:1.63). Vertex (Figs 3, 20) at base wider than length in middle line (1:0.62), apex narrower than base (1:1.45), straightly projecting before eyes, median carina absent, lateral margin distinctly carinate, posterior margin slightly concave. Frons (Fig. 21) moderately narrow, near frontoclypeal suture widest, disc concave, lateral margins broadly concave inward, distinctly carinate, median carina absent. Postclypeus (Fig. 21) with median and lateral carinae, anteclypeus with weak median carina, lateral carinae absent. Apical segment of rostrum longer than wide. Antennae (Figs 20, 22) short, second antennomere oval, flagellum originated from apical point. Subantennal processes (Figs 21, 22) small. Eyes (Figs 21, 22) semicircular; ocelli present, adjacent to eyes. Median length of pronotum short, anterior margin between eyes convex, posterior margin deeply concave, median carina distinct. Mesonotum (Fig. 20) as long as broad, slightly convex, in lateral view raised above vertex, with median carina distinct and lateral carina weak, posterior end triangularly depressed. Forewing (Fig. 23) narrow, 3.3 times as long as the widest point, clavus closed, claval veins with a prominent ridge of tubercles, base of costal margin curved inward, costal margin also granulated. Hindwing (Fig. 24) shorter than forewing. Hind tibia without lateral spine.

*Male genitalia*. Anal tube (Fig. 25) in lateral view, obliquely, slender at basal half, apical margin rounded, anal styles sets at basal one-fifth; in dorsal view (Fig. 26), length in middle line approximately three times as long as wide at middle, asymmetrical, apex rounded. Pygofer (Fig. 25) in lateral view distinctly narrowed medially, processes (Fig. 27) of pygofer asymmetrical, left dorsocaudal process slightly longer than right one. Gonostyli (Fig. 25) bilaterally asymmetrical, right gonostylus larger than left one, large, elongate and slightly reaching less than apex of anal tube in lateral view, inner side with saccate process at basal two-thirds near ventral margin, left gonostylus with a small process rising from apical one-fifth of dorsal margin. Phallus asymmetrical, periandrium curved, with a hooked process near base ventrally directed caudally, in left lateral view (Fig. 28), with a slender process near middle, directed dorsocaudally, and two stout processes at apex, in right lateral view (Fig. 29), with a plate near apex, and a long process at apical two-thirds, slightly curved, directed dorsally, apical margin serrate. Aedeagus with five spinous processes at apex, the largest process produced reaching to base of periandrium, acute at apex.

**Figures 30–34.**
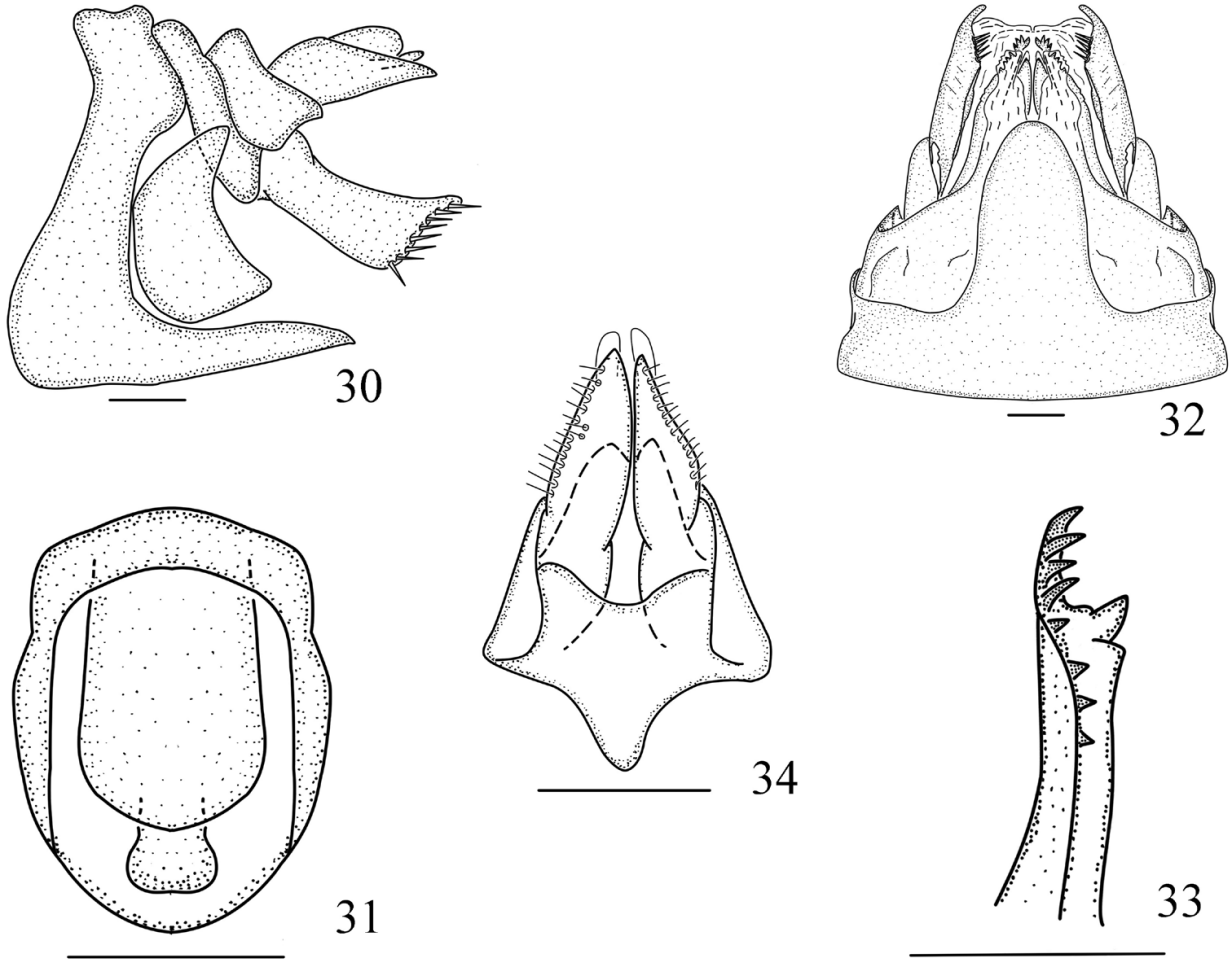
*Vekuntapentaprocessusa* sp. n., female. **30** Genitalia, lateral view **31** anal segment, dorsal view **32** genitalia, ventral view **33** gonapophysis VIII, right lateral view **34** gonapophysis IX, ventral view. Scale bar: 0.2 mm.

*Female genitalia.* Anal tube (Figs 30, 31) symmetrical and ring-shaped in dorsal view; apex of anal tube slightly exceeding apex of anal style. Abdominal sternite VII (Fig. 32) in ventral view symmetrical, posterior margin protruded medially, with protrusion length shorter than width at base, apical margin rounded. Gonapophysis VIII (Figs 32, 33) with eight teeth at ventral margin. Gonapophysis IX (Fig. 34) with two lobes incompletely symmetrical, lateral margin with dense setae, each lobe with a membrane sheet dorsally, blunt apically. Gonoplac (Figs 30, 32) in lateral view nearly rectangular, with a small angulate process at apex dorsally, lateral margin with spiniform setae.

#### Remarks.

This species is similar to *V.fuscolineata*Rahman et al., 2012, but distinguished from the latter by the slightly dark yellow mesonotum (Fig. 20) (mesonotum distinctly dark brown on each side, golden yellow in middle in *V.fuscolineata*); periandrium (Figs 28, 29) with a hooked process near base ventrally, directed caudally (periandrium without process ventrobasally in *V.fuscolineata*); anal tube of male (Fig. 26) asymmetrical in dorsal view (symmetrical in dorsal view in *V.fuscolineata*); gonostyli (Fig. 25) asymmetrical, with right gonostylus distinctly larger than left one in lateral view (symmetrical in lateral view in *V.fuscolineata*).

#### Etymology.

The new species name is derived from the Latin words *penta*- (five) and *processus* (process), referring to the apex of aedeagus with five processes in male.

#### Host plant.

Unknown.

#### Distribution.

China (Yunnan).

## Discussion

The genus *Vekunta* is a diverse genus in the subtropical and tropical regions of Australasian, Oriental, and Palaearctic regions (Löcker et al. 2009, Rahman et al. 2012). To date, there are 43 species recorded in the world (Bourgoin 2018). Approximately 27 species of genus *Vekunta* are known in China, distributed in Zhejiang (one species), Yunnan (one species), Tibet (one species), and Taiwan (twenty-four species). Almost all members of the genus in China are distributed in the Oriental region, hence especially in southern China.

Due to the original literature not recording host plants of this genus, many host plants are unknown. In our study, we find that *V.bambusana* sp. n. lives on bamboo in Guizhou, and some species of *Vekunta* we collected on weeds in some humid environments, for example, *V.triprotrusa* Wu & Liang, 2001. The new species *V.pentaprocessusa* sp. n. was collected by light trap. Thus, we speculate that this group prefers warm and moist environments and some species of the genus *Vekunta* have phototaxis. The natural environment of China is diverse, such as Yunnan Province (southern China), one of China’s richest regions in terms of biodiversity; however, only one species of genus *Vekunta* has been recorded in this region, so we believe there should be more species of this genus waiting to be discovered in this region and other parts of China.

## Supplementary Material

XML Treatment for Vekunta

XML Treatment for Vekunta bambusana

XML Treatment for Vekunta pentaprocessusa
